# Impact of molecular subtypes on metastatic breast cancer patients: a SEER population-based study

**DOI:** 10.1038/srep45411

**Published:** 2017-03-27

**Authors:** Yue Gong, Yi-Rong Liu, Peng Ji, Xin Hu, Zhi-Ming Shao

**Affiliations:** 1 Department of Breast Surgery, Key Laboratory of Breast Cancer in Shanghai, Fudan University Shanghai Cancer Center, Fudan University, Shanghai, 200032, China; 2 Department of Oncology, Shanghai Medical College, Fudan University, Shanghai, 200032, China; 3 Institutes of Biomedical Science, Fudan University, Shanghai, 200032, China

## Abstract

To investigate the significance and impact of molecular subtyping stratification on metastatic breast cancer patients, we identified 159,344 female breast cancer patients in the Surveillance, Epidemiology and End Results (SEER) database with known hormone receptor (HoR) and human epidermal growth factor receptor 2 (HER2) status. 4.8% of patients were identified as having stage IV disease, and were more likely to be HER2+/HoR−, HER2+/HoR+, or HER2−/HoR−. Stage IV breast cancer patients with a HER2+/HoR+ status exhibited the highest median overall survival (OS) (44.0 months) and those with a HER2−/HoR− status exhibited the lowest median OS (13.0 months). Patients with a HER2−/HoR+ status had more bone metastasis, whereas patients with a HER2+/HoR− status had an increased incidence of liver metastasis. Brain and lung metastasis were more likely to occur in women with a HER2−/HoR− status. The multivariable analysis revealed a significant interaction between single metastasis and molecular subtype. No matter which molecular subtype, women who did not undergo primary tumour surgery had worse survival than those who experienced primary tumour surgery. Collectively, our findings advanced the understanding that molecular subtype might lead to more tailored and effective therapies in metastatic breast cancer patients.

Breast cancer is the most frequently diagnosed malignant cancer among women worldwide^1^. It is also the second leading cause of cancer death among US females after lung cancer. Approximately 5–8% of patients have distant metastases at the time of diagnosis, and the 5-year cause-specific survival for these patients is only 24% to 39%^2^. Similar to early-stage breast cancer, metastatic breast cancer is also a highly heterogeneous disease and considered to be incurable. Thus, the primary goals of treatment are to prolong survival and ameliorate symptoms; however, there are many factors that influence the therapeutic efficacy of drugs targeting metastatic breast cancer; the molecular subtype being one of these vital prognostic factors^3^. The hormone receptor (HoR)-positive subtype (either estrogen receptor-(ER) positive or progesterone receptor (PR)-positive) is the most common subtype, which can be subdivided into luminal A and luminal B based on gene expression. Compared with the luminal A and luminal B, the human epidermal growth factor receptor 2 (HER2)-overexpressing (HoR−/HER2+) and triple-negative (HoR−/HER2−) subtypes are known to be more aggressive and have poorer outcomes^4^,^5^. These molecular subtypes have also correlated with a risk of local and regional recurrence^6^ and survival after distant metastasis^7^,^8^,^9^.

The preferential relocation to a site distant from a tumour is of clinical and biological importance. The well-known “seed and soil” theory demonstrates that all types of tumours spread in a non-random and organotropic metastatic pattern, and breast cancer is no exception^10^. Some unique gene signatures may induce breast cancer to invade specific organs. The relationship between molecular subtypes and the patterns of distant relapse has been documented. HoR-positive patients are more likely to have bone metastases^11^,^12^, whereas HoR−/HER2+ and HoR−/HER2− patients present more visceral metastases, including to the liver and lung^8^,^9^,^13^. Moreover, patients with bone metastases may have a longer overall survival than those with visceral metastases^14^,^15^.

Traditionally, systemic therapy is the primary treatment of metastatic breast cancer and includes endocrine therapy, chemotherapy, and targeted therapy. Locoregional treatment such as surgical resection of the primary tumour has been used only to control pain or bleeding. Based on the National Comprehensive Cancer Network (NCCN) guidelines, primary tumour surgery should be considered for patients with metastatic breast cancer who either require symptomatic relief or have impending complications; furthermore, this procedure should only be undertaken if complete local clearance of tumour is available and other disease sites are not immediately life-threatening^16^. However, controversy still exists about which subgroup (if any) of metastatic breast cancer patients should undergo primary tumour surgery. Two recent meta-analysis based on several retrospective studies indicated that removing the primary tumour offers a survival benefit, with pooled hazard ratios (HR) for overall mortality of 0.65 (95% confidence interval (CI) = 0.59–0.72)^17^ and 0.69 (95% CI = 0.63–0.77)^18^, whereas a prospective study from India reported no evidence to suggest that locoregional treatment (including surgery and postoperative adjuvant radiation) of the primary tumour affects overall survival (locoregional treatment group vs. no-locoregional treatment group, 19.2 vs. 20.5 months; HR = 1.04, 95% CI = 0.81–1.34)^19^. In addition, whether different molecular subtypes of metastatic breast cancer affect the efficacy of primary tumour surgery is still unknown.

The objective of this study is to demonstrate the significance and impact of the molecular subtype on metastatic breast cancer patients’ survival, site of distant metastasis and effect of primary tumour surgery. We utilized Surveillance, Epidemiology, and End Results (SEER) population-based data to perform high-powered statistical analysis. Through this, we developed a deeper understanding of the relationship between stage IV breast cancer and the patients’ HoR and HER2 status.

## Results

### Patient characteristics

The demographic and clinical characteristics of the study cohort based on breast cancer stage are shown in Table 1. Of the 159,344 female breast cancer patients included in the analysis, 151,766 patients (95.2%) were diagnosed with stage I–III breast cancer, whereas 7,578 women (4.8%) were stage IV breast cancer. Compared with the stage I–III group, stage IV patients had larger tumours (tumours >5 cm in size: 36.7% vs 8.0%, for stage IV and stage I–III respectively) and more advanced disease (grade III and undifferentiated (UD): 44.2% vs 32.4%). More stage IV women were categorized with HER2+/HoR− (9.2% vs 4.5%), HER2+/HoR+ (17.1% vs 10.5%) and HER2−/HoR− (13.2% vs 11.4%) status compared with stage I–III patients. Fewer primary tumour surgery (37.3% vs 96.4%) and radiation (34.5% vs 52.6%) were used to treat stage IV breast cancer patients than stage I–III patients. The demographic and pathological features of the metastatic breast cancer patients based on the HoR/HER2 phenotypes are summarized in Table S1.

### Impact of molecular subtype on the survival outcomes of stage IV patients

Kaplan-Meier analysis was used to determine overall survival (OS) and breast cancer-specific survival (BCSS) in the groups based on stage at the time of diagnosis (Figure S1A,B). As expected, stage IV patients exhibited worse survival rates than stage I–III patients (P < 0.001). To determine prognostic factors of stage IV breast cancer patients, we used univariate and multivariate Cox proportional hazard models to analyse the data (Table 2). In the univariate analysis, age, race, insurance status, marital status, tumour grade, and history of primary tumour surgery and/or radiation were significantly associated with OS and BCSS (P < 0.05). The multivariate analysis of stage IV patients was adjusted for all the prognostic factors listed in the Table 2 and age, marital status, tumour grade, molecular subtype and history of primary tumour surgery were identified as independent prognostic factors for both OS and BCSS. Besides, compared with white women, black women with stage IV breast cancer had worse prognosis while other race of patients seemed no difference in the prognosis. Insurance status was significantly associated with BCSS but not with OS, but year of diagnosis, tumour size, number of positive regional nodes and radiation were not correlated with the prognosis of stage IV patients.

**Figure 1:**
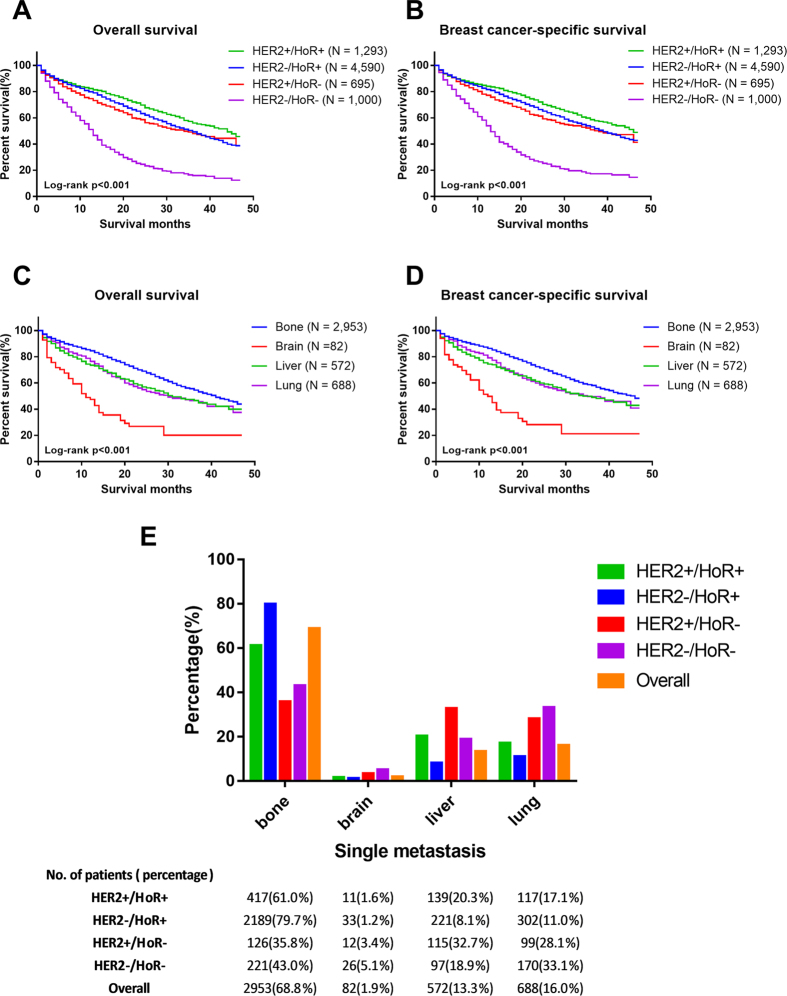
(**A,B**) Overall and breast cancer-specific survival of stage IV patients according to molecular subtype. (**C**,**D**) Overall and breast cancer-specific survival of stage IV patients with single metastasis according to site of distant metastasis. (**E**) Distribution of single metastasis in stage IV patients with different molecular subtypes. P-value of all survival curves was less than 0.001.

When we focused on the relationship between molecular subtype and prognosis of stage IV patients, we observed that stage IV patients with a HER2+/HoR+ status exhibited the prolonged OS, whereas patients with a HER2−/HoR− status exhibited the worst OS (Fig. 1A). The median OS of the entire stage IV cohort was 32.0 months (95% CI: 30.5–33.5 months). The median survival ranged from 13.0 months (95% CI: 12.2–13.8 months) for patients with the HER2−/HoR− subtype to 44.0 months (95% CI: undetermined) for patients with the HER2+/HoR+ subtype (P < 0.001). Patients with HER2−/HoR+ (median OS = 36.0 months, 95% CI: 34.1–37.9 months) and HER2+/HoR− status (median OS = 34.0 months, 95% CI 27.4–40.6 months) presented similar OS rates but exhibited better survival compared with HER2−/HoR− patients (P < 0.001). Similar results were observed for BCSS (Fig. 1B). These results were essentially consistent with the abovementioned multivariate analysis.

### Relationship between molecular subtypes and site of distant metastasis

Among the 7,578 stage IV patients, 4,295 women had data for a single metastasis, which indicated that they had only one site of distant metastasis to the bone, brain, liver or lung. Figure 1E illustrated that stage IV patients with a HER2−/HoR+ status had more bone metastasis (HER2−/HoR+ vs HER2+/HoR− vs HER2+/HoR+ vs HER2−/HoR−: 79.7% vs 35.8% vs 61.0% vs 43.0%, respectively), whereas patients with a HER2+/HoR− status had an increased incidence of liver metastasis (HER2−/HoR+ vs HER2+/HoR− vs HER2+/HoR+ vs HER2−/HoR−: 8.1% vs 32.7% vs 20.3% vs 18.9%, respectively). Brain metastasis (HER2−/HoR+ vs HER2+/HoR− vs HER2+/HoR+ vs HER2−/HoR−: 1.2% vs 3.4% vs 1.6% vs 5.1%, respectively) and lung metastasis (HER2−/HoR+ vs HER2+/HoR− vs HER2+/HoR+ vs HER2−/HoR−: 11.0% vs 28.1% vs 17.1% vs 33.1%, respectively) were more likely to occur in women with a HER2−/HoR− status. In general, bone metastasis ranked as the most common site of distant metastasis among stage IV breast cancer patients (68.8%), followed by lung metastasis (16.0%), liver metastasis (13.3%) and brain metastasis (1.9%).

### Impact of single metastasis on the prognosis of stage IV patients

In contrast, patients with bone metastasis (median OS = 41.0 months, 95% CI: 38.0–44.0 months) exhibited the best survival rates, whereas those with brain metastasis (median OS = 11.0 months, 95% CI: 7.5–14.5 months) exhibited the worst survival rates with regard to both OS and BCSS (P < 0.001). Patients with liver metastasis (median OS = 31.0 months, 95% CI: 25.5–36.5 months) and lung metastasis (median OS = 30.0 months, 95% CI: 23.6–36.4 months) appear to experience similar survival rates (Fig. 1C,D). Individual survival curves for the four molecular subtypes according to site of distant metastasis were also generated (Figure S2A–H), which showed OS and BCSS of stage IV patients with HER2−/HoR+ or HER2+/HoR− subtype had no differences according to site of distant metastasis (P < 0.05), whereas there were no statistical differences in the HER2+/HoR+ and HER2−/HoR− subgroups (P > 0.05). When single metastasis was included as a prognostic factor in the Cox proportional hazards regression model analysis (Table S2), we found that the HR observed in the multivariate analysis was piecewise. When used bone metastasis as reference, brain (HR = 2.57, 95% CI = 1.92 to 3.44 in the OS cohort; HR = 2.71, 95% CI = 2.01 to 3.66 in the BCSS cohort) and liver metastasis (HR = 1.40, 95% CI = 1.20 to 1.65 in the OS cohort; HR = 1.45, 95% CI = 1.22 to 1.71 in the BCSS cohort) had lower HRs whereas the HR was not significantly different in the lung metastasis group (HR = 1.00, 95% CI = 0.86 to 1.16 in the OS cohort; HR = 1.00, 95% CI = 0.85 to 1.17 in the BCSS cohort).

### Primary tumour surgery and survival of stage IV patients

**Figure 2:**
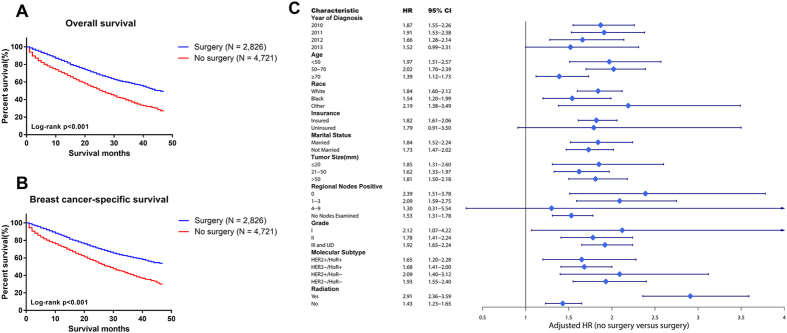
(**A**,**B**) Overall and breast cancer-specific survival of stage IV patients who received primary tumor surgery or not. P-value of all survival curves was less than 0.001. (**C**) Forest plot of multivariate analysis for overall survival of stage IV patients with surgery or no surgery using the Cox regression model by adjusting for all other prognostic factors listed. The diamond denotes the HR of each subgroup. An HR > 1.0 indicates higher risk for overall mortality in the no surgery group and vice versa.

We found that the hazard ratio in stage IV patients without a history of primary tumour surgery was higher than that of those who underwent primary tumour surgery (HR = 1.80, 95% CI = 1.60 to 2.02 in the OS cohort; HR = 1.81, 95% CI = 1.60 to 2.05 in the BCSS cohort). Furthermore, we used Kaplan-Meier analysis to determine the OS and BCSS between the two subgroups. We found that patients who underwent primary tumour surgery had better OS and BCSS compared with those not undergoing primary tumour surgery (Fig. 2A,B). Upon analysis of the molecular subtype subgroup, the results were the same (Figure S3A–H). Moreover, the prognostic significance of the history of primary tumour surgery persisted in each subgroup when stratified by other prognostic factors (Fig. 2C). With regard to the molecular subtype subgroups, we found that no matter which molecular subtype, women who did not undergo primary tumour surgery had worse survival than those who experienced primary tumour surgery (HR = 1.65, 95% CI = 1.20 to 2.28 in the HER2+/HoR+ cohort; HR = 1.68, 95% CI = 1.41 to 2.00 in the HER2−/HoR+ cohort; HR = 2.09, 95% CI = 1.40 to 3.12 in the HER2+/HoR− cohort; HR = 1.93, 95% CI = 1.55 to 2.40 in the HER2−/HoR− cohort).

## Discussion

This study analysed recently available data on the HoR and HER2 status in metastatic breast cancer patients from the SEER registries, in an attempt to identify differences in the influence of the breast cancer subtype on the patient prognosis, site of distant metastasis and the effect of primary tumour surgery. In this large retrospective study, we show that the molecular subtype is an independent prognostic factor and is correlated with distant metastasis and the effects of primary tumour surgery.

In accordance with previous data^2^, we reported that 4.8% of breast cancer patients included in the analysis were stage IV and that their survival is much shorter than that of stage I–III breast cancer patients. Compared with stage I–III, stage IV women were more likely to be HER2+/HoR−, HER2+/HoR+, or HER2−/HoR−, and these three subgroups comprised 40% of the patients with metastatic breast cancer while only representing 26% of case patients with stage I–III breast cancer. This finding has been previously reported and is consistent with the increased aggressiveness of these tumour subtypes compared with that of HER2−/HoR+ disease^3^,^20^.

Our results indicate that the prognosis among the different molecular subtypes of stage IV breast cancer patients is highly variable. HER2+/HoR+ patients had the best survival among the four subgroups. The median OS and BCSS of HER2+/HoR+ patients is approximately 3.5-fold that of HER2−/HoR− patients, whereas HER2−/HoR+ patients and HER2+/HoR− patients exhibited similar median survival rates. This result is somewhat contrary with that of a recent study on metastatic breast cancer subtypes^8^, which included 3,726 patients with early-stage breast cancer diagnosed between 1986 and 1992 with archival tissue; this report indicated that 1,357 patients developed distant metastases during the follow-up period. The median survival durations among patients with distant metastasis were largely different among each subtype. The luminal/HER2-positive (median survival = 1.3 years, 95% CI: 1.1–1.7 years) and HER2-enriched (median survival = 0.7 years, 95% CI: 0.6–0.8 years) subtypes had significantly lower median survival rates compared with that of the luminal A (median survival = 2.2 years, 95% CI: 1.9–2.5 years) and luminal B (median survival = 1.6 years, 95% CI: 1.4–1.8 years) subtypes. The reason for this discrepancy may be that there was no HER2-targeted therapy for metastatic breast cancer prior to 1998. After the development of HER2-targeted therapies such as trastuzumab, pertuzumab, lapatinib and T-DM1 for the treatment of metastatic breast cancer, the survival of HER2-positive patients was greatly increased^7^,^9^,^21^. For HER2+/HoR+ patients, the therapeutic strategy for metastatic breast cancer includes both endocrine therapy and HER2-targeted therapy, which may prolong the survival time. In general, our study confirmed that compared with other primary tumour characteristics, the molecular subtypes based on the HoR and HER2 status of the primary tumour could be of significant prognostic relevance for survival.

It is well known that site of distant metastasis is related to the survival of stage IV breast cancer patients. As previously reported^9^,^14^,^22^, we found that patients with bone metastasis showed the best prognosis, and patients with brain metastasis showed worst prognosis. One of the reasons why women with brain metastasis had an unfavourable prognosis is because many systemic therapies fail to cross the blood brain barrier, and treatment options for brain metastasis are particularly limited^23^. There is also a theory that once a metastasis breaches the blood brain barrier, a different blood tumour barrier can form, which in turn might lead to limited drug delivery^24^. Fortunately, because of increased understanding of the blood brain barrier and the development of tyrosine kinase inhibitors and monoclonal antibodies, some novel agents are being investigated as treatment options for metastatic breast cancer patients with brain metastasis^25^. We also discovered that in the HER2−/HoR+ and HER2+/HoR− subgroups, the survival of patients with brain metastasis was still the worst but in the HER2+/HoR+ and HER2−/HoR− subgroups, there were no differences between the survival of patients with brain metastasis and that of patients with other sites of distant metastasis. This may be because the HER2−/HoR− subtype breast cancer is an inherently aggressive malignant disease with a lack of recognized therapeutic targets; thus, regardless of the target organ for the primary tumour metastasis, there are few effective treatments for patients^26^. Moreover, the number of patients with brain metastasis is much smaller than that of patients with other metastasis and the results may be more convincing if more patients are included into analysis.

In our study, we reported that the frequency of the first site of distant metastasis differed among the molecular subtypes. As expected, HER2−/HoR+ patients presented with the highest frequency of bone metastatic disease, which consisted in 79.7% of the HER2−/HoR+ cases^9^. The strong association of hormonal receptor status with bone metastasis was proposed early in 1991^27^. With a deeper understanding of the modulated genes and pathways in the various subgroups, it has become more evident that bone metastasis is most abundant among the hormonal receptor-positive subtypes^12^. In the current study, the HER2+/HoR− and HER2/HoR− subtypes exhibited more metastasis to the brain alone than the other two molecular subtypes^8^,^28^. The HER2 status has been reported to have a strong relationship with brain metastasis, and HER2-positive breast cancer has a potential affinity for brain tissue. A preclinical study indicated that HER2 overexpression increased the outgrowth of metastatic breast tumour cells in the brain *in vivo*^29^, and another study revealed that the blood brain barrier was likely preserved in the brain metastases of HER2-positive breast cancer^30^. Active Wnt/β-catenin signaling has also been found to exert some effect on HER2−/HoR− tumours that metastasize to the brain^31^. Moreover, liver only metastasis was most frequent in HER2+/HoR− patients, whereas lung only metastasis was most common in HER2−/HoR− patients. There are some studies that can explain this phenomenon. CXCR4, a chemokine receptor enhanced by HER2 activation, has been proposed to be involved in promoting the invasion of tumour cells to liver^32^, and the focal adhesion signaling cascade, which is down-regulated in HER2−/HoR− patients, is an important modulator of lung-specific relapse^12^.

Our investigation includes molecular subtypes as a prognostic factor and provides evidence of a clear association of primary tumour surgery in stage IV patients with increased OS and BCSS. Similar to our research, several analysis have also reported that primary tumour surgery has a favourable prognostic impact in stage IV breast cancer patients^17^,^18^,^33^. We used a Cox proportional regression model by adjusting for all the prognostic factors and demonstrated an obvious benefit of primary tumour removal. HER2+/HoR− subtype patients appear to achieve more benefits from primary tumour surgery (HR = 2.09, 95% CI: 1.40–3.12) than patients with any other molecular subtype. In addition, regardless of the subtype, patients who underwent surgery had better survival than those who did not. This finding reminds us that removing the primary tumour may suppress further tumour spread or reverse tumour-induced immunosuppression^34^.

Some limitations in our study should be mentioned. One is that the subtypes were based on the HoR and HER2 status without incorporating other markers such as Ki-67, which is an important index to distinguish the luminal A and luminal B subtypes. This may contribute to some disparities between our investigation and clinical applications. Additionally, histological biopsies of the metastatic lesions were not included in the analysis. Re-evaluating the HoR and HER2 statuses was recommended in a recent international consensus of guidelines for advanced breast cancer^35^. Thus, more analysis is necessary to determine the impact of altering the HoR and HER2 statuses on the effectiveness of systemic treatment. Furthermore, the SEER database does not provide any information on systemic treatments such as endocrine therapy, HER2-targeted therapy or chemotherapy. Therefore, these prognostic factors cannot be obtained and adjusted for the observed results, thus causing deviations in the analysis. In addition, although the multivariate analysis were conducted to reduce the confounding factors, any bias due to the imbalance of the surgery group compared with the no surgery group cannot be totally excluded.

In conclusion, our study supports the impact of molecular subtypes on stage IV breast cancer patients, site of distant metastasis and effects of primary tumour surgery. Each molecular subtype has its own biological characteristics and exerts different activities in promoting metastatic breast cancer. Although further investigation on the gene modulation and molecular mechanism of the different molecular subtypes are desirable, currently available evidence should be discussed with patients.

## Methods

### Study population

We obtained data from the current SEER database (November 2015 Submission) which consists of 18 population-based cancer registries, covering approximately 28% of the total population of the United States. SEER data are an open access resource for cancer-based demographic and clinical information as well as treatment and patient survival. SEER*Stat Version 8.3.2 (http://www.seer.cancer.gov/seerstat) from the National Cancer Institute (NCI) was used to identify eligible patients.

**Figure 3:**
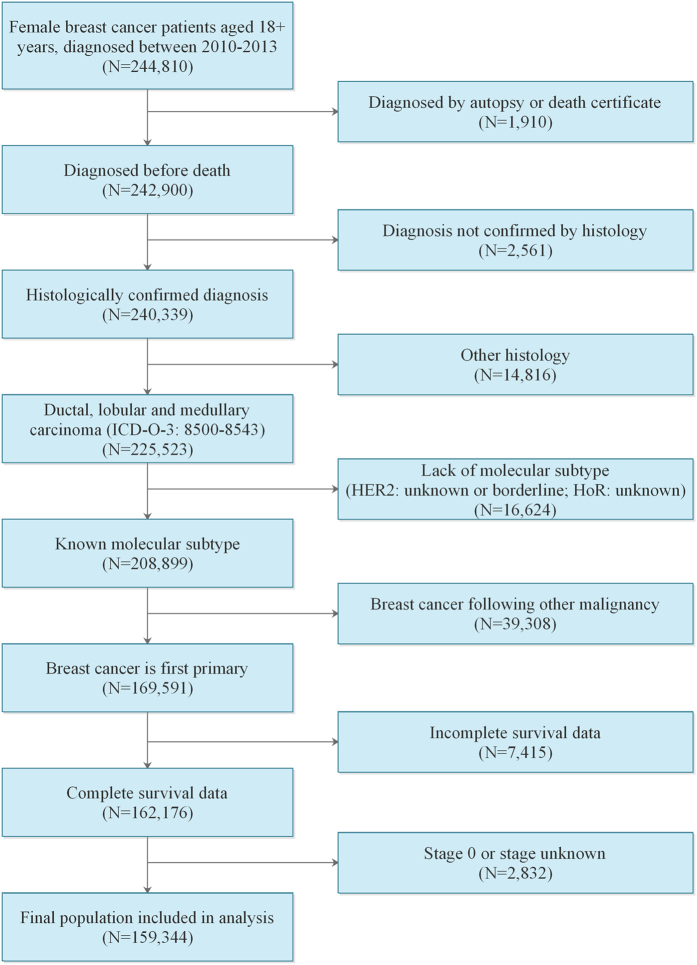
Flow diagram for selection of the study cohort.

Because the SEER database began collecting information on the HER2 status and sites of distant metastasis in 2010, we included adult women (≥18 years of age) diagnosed between 2010 and 2013, which totaled 244,810 initial cases. Patients diagnosed by either autopsy or death certificate were excluded. The analysis was restricted to a diagnosis confirmed by histopathology, and only ductal, lobular and medullary carcinomas based on the primary site were included (International Classification of Disease for Oncology, Third Edition (ICD-O-3) codes 8500 to 8543). The ER and PR results were combined as the HoR status, and patients with a borderline ER or PR status were defined as HoR+, whereas patients with a borderline HER2 status were defined as having an unknown HER2 status. The breast cancer molecular subtype was stratified based on joint HoR and HER2 statuses (HER2−/HoR+, HER2+/HoR−, HER2+/HoR+, HER2−/HoR−). We excluded patients whose molecular subtype was unknown (either HoR status was unknown or HER2 status was unknown or borderline). Patients with secondary malignancies at the time of breast cancer diagnosis (n = 39,308), patients with incomplete survival data (n = 7,415), or patients with American Joint Committee on Cancer (AJCC) stage 0 or unknown staging (n = 2,832) were also excluded. In total, 159,344 women were eventually eligible for inclusion in the present analysis (Fig. 3).

This study was conducted with approval from the Ethical Committee Review Board of Fudan University Shanghai Cancer Center and determined to not be human participant research; therefore, it does not require informed consent.

### Statistical analysis

Descriptive statistics were used to examine the following baseline characteristics of the breast cancer patients: year of diagnosis, age, race/ethnicity, insurance status, marital status, tumour size, regional node status, grade, molecular subtype, surgery and radiation. These variables were stratified by breast cancer stage at the time of diagnosis. P-values for comparing the frequency distributions among the subgroups were calculated using the chi-squared (x2) test.

BCSS and OS were used as the primary study outcomes, and BCSS was defined as the time from the breast cancer diagnosis to death due to breast cancer and OS as the time from the breast cancer diagnosis to death due to any cause. We used the Kaplan-Meier method to generate survival curves and analysed the differences between the curves using the log-rank test. Univariate and multivariate Cox proportional hazard models were applied to identify risk factors for BCSS and OS, and the HRs and 95% CIs were reported.

All of the statistical analysis were performed using the R statistical software, version 3.3.1 (www.r-project.org) and SPSS statistical software, version 22.0 (IBM Corp, Armonk, NY). A two-sided P-value less than 0.05 was considered statistically significant.

## Additional Information

**How to cite this article:** Gong, Y. *et al*. Impact of molecular subtypes on metastatic breast cancer patients: a SEER population-based study. *Sci. Rep.* **7**, 45411; doi: 10.1038/srep45411 (2017).

**Publisher's note:** Springer Nature remains neutral with regard to jurisdictional claims in published maps and institutional affiliations.

## Supplementary Material

Supplementary Information

## Figures and Tables

**Table 1:** Demographic and clinical characteristics of the study cohort.

	Stage I–III	Stage IV	Total	p-value^a^
	N = 151,766(%)	N = 7,578(%)	N = 159,344(%)	
Year of Diagnosis				0.202
2010	36,438(24.0)	1,791(23.6)	38,229(24.0)	
2011	38,485(25.4)	1,924(25.4)	40,409(25.4)	
2012	39,618(26.1)	1,928(25.4)	41,546(26.1)	
2013	37,225(24.5)	1,935(25.5)	39,160(24.6)	
Age				0.658
<50	35,510(23.4)	1,806(23.8)	37,316(23.4)	
50–69	78,931(52.0)	3,929(51.8)	82,860(52.0)	
≥70	37,325(24.6)	1,843(24.3)	39,168(24.6)	
Race				<0.001
White	120,366(79.3)	5,670(74.8)	126,036(79.1)	
Black	16,536(10.9)	1,291(17.0)	17,827(11.2)	
Others^b^	13,898(9.2)	587(7.7)	14,485(9.1)	
Unknown	966(0.6)	30(0.4)	996(0.6)	
Insurance				
Insured	146,906(96.8)	7,092(93.6)	153,998(96.6)	<0.001
Uninsured	2,719(1.8)	349(4.6)	3,068(1.9)	
Unknown	2,141(1.4)	137(1.8)	2,278(1.4)	
Marital status				
Married	85,110(56.1)	3341(44.1)	88,451(55.5)	<0.001
Not married^c^	59,011(38.9)	3833(50.6)	62,844(39.4)	
Unknown	7,645(5.0)	404(5.3)	8,049(5.1)	
Tumor size(mm)				<0.001
≤20	85,669(56.4)	924(12.2)	86,593(54.3)	
21–50	53,352(35.2)	2,895(38.2)	56,247(35.3)	
>50	12,194(8.0)	2,784(36.7)	14,978(9.4)	
Unknown	551(0.4)	975(12.9)	1,526(1.0)	
Regional nodes positive				<0.001
0	95,906(63.2)	457(6.0)	96,363(60.5)	
1–3	31,644(20.9)	1,000(13.2)	32,644(20.5)	
4–9	8566(5.6)	573(7.6)	9,139(5.7)	
>10	3969(2.6)	512(6.8)	4,481(2.8)	
No nodes examined	9,361(6.2)	3,885(51.3)	13,246(8.3)	
Unknown	2,320(1.5)	1,151(15.2)	3,471(2.2)	
Grade				<0.001
I	32,636(21.5)	496(6.5)	33,132(20.8)	
II	65,133(42.9)	2,791(36.8)	67,924(42.6)	
III and UD^d^	49,211(32.4)	3,352(44.2)	52,563(33.0)	
Unknown	4,786(3.2)	939(12.4)	5,725(3.6)	
Molecular subtype				<0.001
HER2+/HoR+	15,897(10.5)	1,293(17.1)	17,190(10.8)	
HER2−/HoR+	111,865(73.7)	4,590(60.6)	116,455(73.1)	
HER2+/HoR−	6,762(4.5)	695(9.2)	7,457(4.7)	
HER2−/HoR−	17,242(11.4)	1,000(13.2)	18,242(11.4)	
Surgery				<0.001
Yes	146,276(96.4)	2,826(37.3)	149,102(93.6)	
No	5,440(3.6)	4,721(62.3)	10,161(6.4)	
Unknown	50(0.0003)	31(0.4)	81(0.0005)	
Radiation				<0.001
Yes	79,890(52.6)	2,613(34.5)	82,503(51.8)	
No	65,422(43.1)	4,726(62.4)	70,148(44.0)	
Unknown	6,454(4.3)	239(3.2)	6693(4.2)	

Abbreviations: UD, undifferentiated; HER2, human epidermal growth factor receptor-2; HoR, hormone receptor.

^a^p-value was assessed using the Pearson’s χ2 test.

^b^Including American Indian/Alaskan native and Asian/Pacific Islander.

^c^Including divorced, separated, single (never married), and widowed.

^d^Including grade 3 and undifferentiated.

**Table 2:** Cox proportional hazards regression model analysis of overall mortality and breast cancer-specific mortality of stage IV breast cancer patients.

	Overall mortality				Breast cancer-specific mortality			
	Univariate analysis		Multivariate analysis		Univariate analysis		Multivariate analysis	
	HR (95% CI)	P-value	HR (95% CI)	P-value	HR (95% CI)	P-value	HR (95% CI)	P-value
Year of Diagnosis								
2010	Reference	—	Reference	—	Reference	—	Reference	—
2011	0.98(0.89–1.07)	0.594	0.97(0.88–1.06)	0.484	0.99(0.90–1.09)	0.903	0.99(0.89–1.08)	0.754
2012	0.92(0.83–1.03)	0.149	0.94(0.84–1.04)	0.236	0.94(0.84–1.05)	0.271	0.95(0.85–1.07)	0.405
2013	0.85(0.73–0.99)	0.034	0.81(0.70–0.94)	0.006	0.87(0.74–1.02)	0.076	0.83(0.71–0.97)	0.017
Age								
<50	Reference	—	Reference	—	Reference	—	Reference	—
50–69	1.30(1.18–1.44)	<0.001	1.26(1.14–1.39)	<0.001	1.27(1.14–1.40)	<0.001	1.22(1.11–1.36)	<0.001
≥70	2.10(1.89–2.34)	<0.001	1.97(1.76–2.20)	<0.001	1.91(1.70–2.13)	<0.001	1.80(1.60–2.02)	<0.001
Race								
White	Reference	—	Reference	—	Reference	—	Reference	—
Black	1.31(1.19–1.44)	<0.001	1.17(1.06–1.29)	0.002	1.30(1.17–1.43)	<0.001	1.14(1.03–1.27)	0.009
Others^a^	0.82(0.70–0.95)	0.009	0.90(0.77–1.05)	0.184	0.82(0.70–0.96)	0.014	0.89(0.76–1.04)	0.143
Insurance								
Insured	Reference	—	Reference	—	Reference	—	Reference	—
Uninsured	1.28(1.07–1.52)	0.006	1.19(1.0–1.42)	0.051	1.35(1.13–1.61)	0.001	1.23(1.03–1.47)	0.025
Marital status								
Married	Reference	—	Reference	—	Reference	—	Reference	—
Not married^b^	1.48(1.37–1.60)	<0.001	1.27(1.17–1.38)	<0.001	1.44(1.33–1.56)	<0.001	1.27(1.15–1.36)	<0.001
Tumor size(mm)								
≤20	Reference	—	Reference	—	Reference	—	Reference	—
21–50	0.89(0.78–1.01)	0.067	0.91(0.80–1.03)	0.139	0.91(0.79–1.04)	0.144	0.93(0.81–1.06)	0.260
>50	1.19(1.05–1.34)	0.007	1.10(0.98–1.25)	0.119	1.24(1.09–1.41)	0.001	1.15(1.01–1.31)	0.039
Regional nodes positive								
0	Reference	—	Reference	—	Reference	—	Reference	—
1–3	1.14(0.92–1.40)	0.240	1.13(0.91–1.40)	0.280	1.14(0.91–1.42)	0.243	1.14(0.91–1.42)	0.264
4–9	1.06(0.84–1.34)	0.634	1.31(1.03–1.66)	0.025	1.05(0.82–1.35)	0.680	1.31(1.02–1.67)	0.035
>10	1.14(0.90–1.44)	0.296	1.42(1.12–1.80)	0.004	1.15(0.90–1.48)	0.266	1.46(1.13–1.87)	0.003
No nodes exmamined	2.14(1.78–2.57)	<0.001	1.59(1.30–1.95)	<0.001	2.15(1.77–2.60)	<0.001	1.61(1.30–2.00)	<0.001
Grade								
I	Reference	—	Reference	—	Reference	—	Reference	—
II	1.33(1.11–1.60)	0.002	1.30(1.08–1.56)	0.006	1.40(1.15–1.70)	0.001	1.36(1.11–1.65)	0.003
III and UD^c^	1.91(1.60–2.29)	<0.001	1.83(1.52–2.21)	<0.001	2.03(1.68–2.47)	<0.001	1.91(1.57–2.33)	<0.001
Molecular Subtype								
HER2+/HoR+	0.82(0.73–0.92)	0.001	0.83(0.74–0.93)	0.002	0.83(0.73–0.94)	0.002	0.83(0.74–0.94)	0.003
HER2−/HoR+	Reference	—	Reference	—	Reference	—	Reference	—
HER2+/HoR−	1.13(0.99–1.29)	0.072	1.19(1.04–1.36)	0.014	1.14(0.99–1.31)	0.072	1.18(1.02–1.37)	0.023
HER2−/HoR−	2.72(2.48–2.99)	<0.001	2.80(2.53–3.10)	<0.001	2.83(2.57–3.12)	<0.001	2.90(2.60–3.22)	<0.001
Surgery								
Yes	Reference	—	Reference	—	Reference	—	Reference	—
No	1.90(1.75–2.06)	<0.001	1.80(1.60–2.02)	<0.001	1.90(1.74–2.07)	<0.001	1.81(1.60–2.05)	<0.001
Radiation								
Yes	Reference	—	Reference	—	Reference	—	Reference	—
No	1.28(1.18–1.38)	<0.001	1.06(0.98–1.15)	0.157	1.25(1.15–1.36)	<0.001	1.04(0.96–1.14)	0.320

Abbreviations: HR, hazard ratio; CI, confidence interval; UD, undifferentiated; HER2, human epidermal growth factor receptor-2; HoR, hormone receptor.

^a^Including American Indian/Alaskan native and Asian/Pacific Islander.

^b^Including divorced, separated, single (never married), and widowed.

^c^Including grade 3 and undifferentiated.
