# Supplementary material for: Impact of molecular subtypes on metastatic breast cancer patients: a SEER population-based study
Source: Sci Rep. 2017 Mar 27;7:45411. doi: 10.1038/srep45411 (PMC5366953; doi:10.1038/srep45411)
Supplement: Supplementary Information [file srep45411-s1.pdf]

SUPPLEMENTARY INFORMATION

Impact of molecular subtypes on metastatic breast cancer patients: a SEER population-based study

Yue Gong[1,2], Yi-Rong Liu[1,2], Peng Ji[1,2], Xin Hu[1,*], Zhi-Ming, Shao[1,2,3,*]

[1]Department of Breast Surgery, Key Laboratory of Breast Cancer in Shanghai, Fudan University Shanghai Cancer Center, Fudan University, Shanghai, 200032, China

[2]Department of Oncology, Shanghai Medical College, Fudan University, Shanghai, 200032, China

[3]Institutes of Biomedical Science, Fudan University, Shanghai, 200032, China

[*]Corresponding authors: Xin Hu, e-mail: xinhu@fudan.edu.com; Zhi-Ming Shao, e-mail: zhimingshao@yahoo.com

- 3 Supplementary Figures
- 2 Supplementary Tables

Supplementary Figure S1

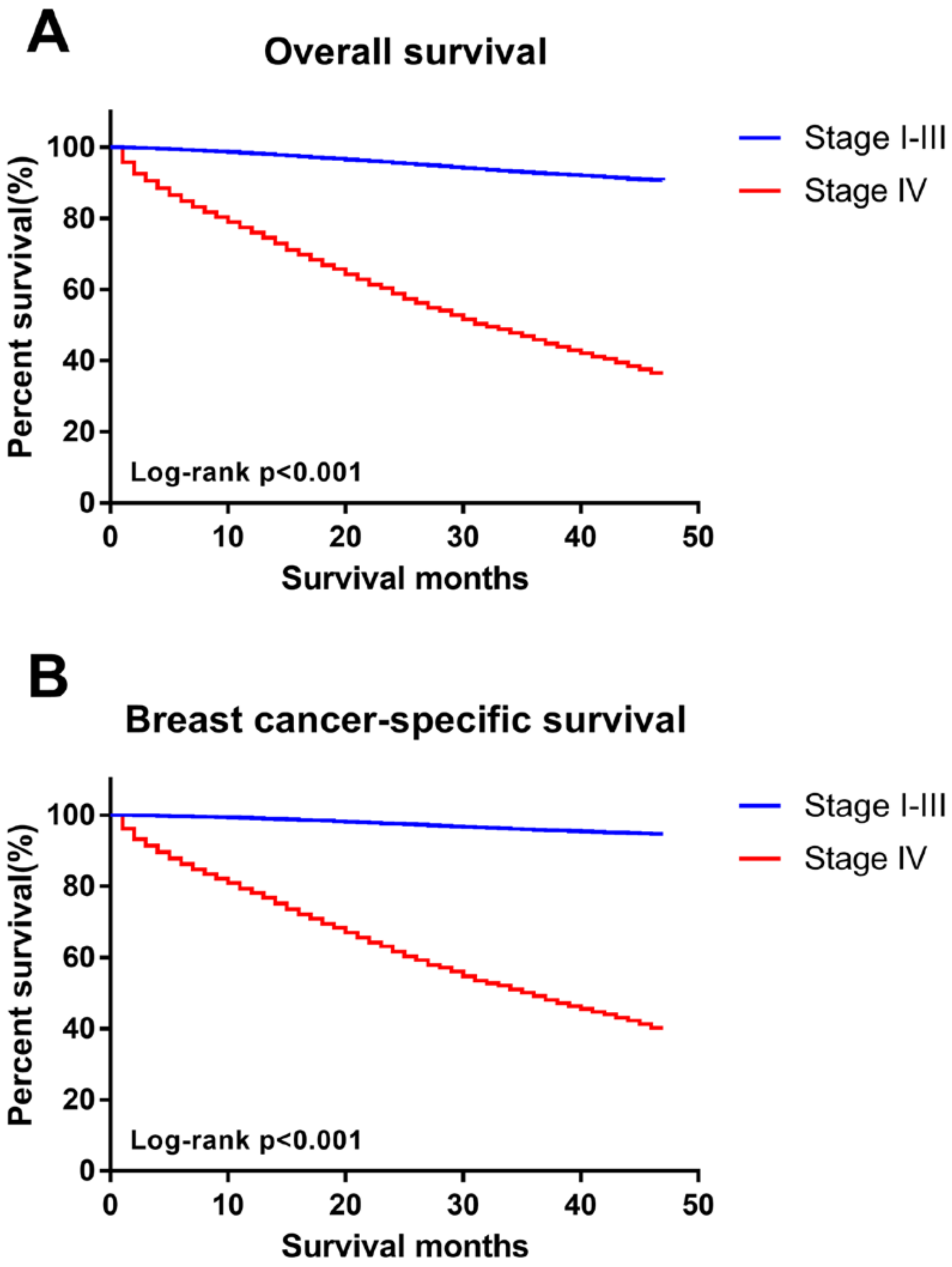

Supplementary Figure S1. A. Overall survival of patients by breast cancer stage. **B.** Breast cancer-specific survival of patients by breast cancer stage.

Supplementary Figure S2

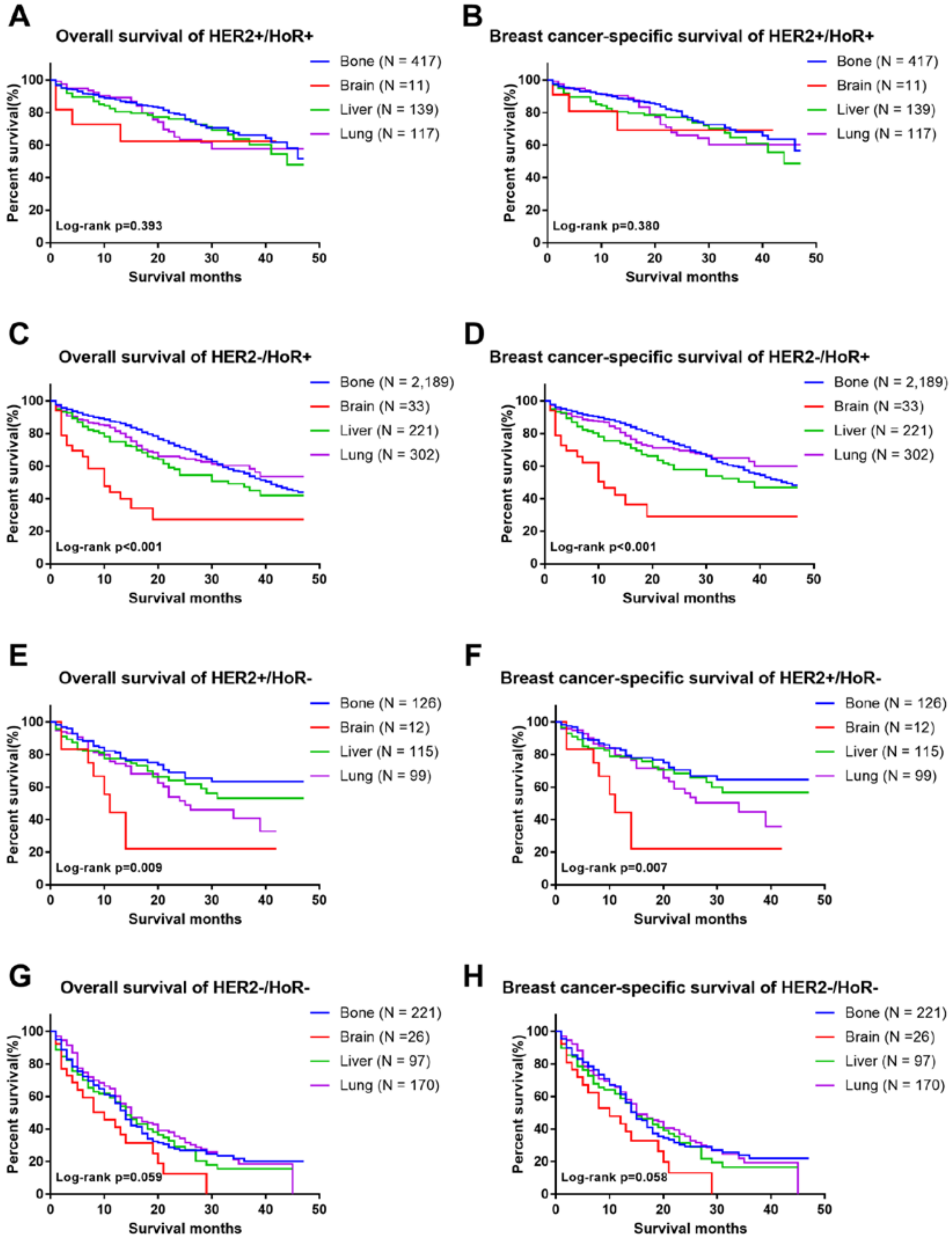

Supplementary Figure S2. A. B. G. and H. Overall survival and breast cancer-specific survival of stage IV patients with HER2+/HoR+ or HER2-/HoR- subtype had no differences according to site of distant metastasis ($P > 0.05$). **C. D. E. and F.** Overall survival and breast cancer-specific survival of stage IV patients with HER2-/HoR+ or HER2+/HoR- subtype had significant differences according to site of distant metastasis ($P < 0.05$).

Supplementary Figure S3

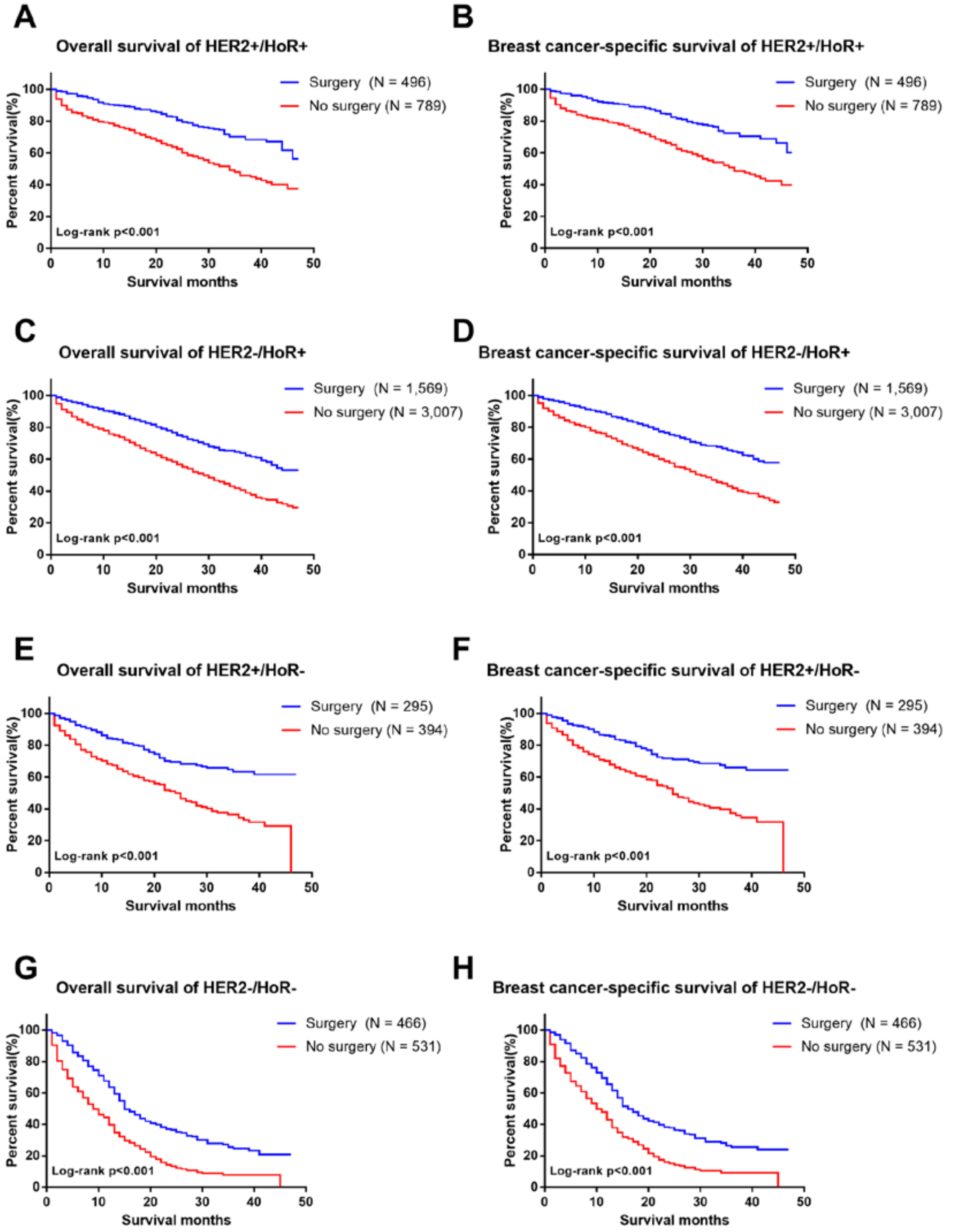

Supplementary Figure S3. A. C. E. and G. Overall survival of stage IV patients with different molecular subtypes according to situation of primary tumor surgery (P < 0.001). **B. D. F. and H.** Breast cancer-specific survival of stage IV patients with different molecular subtypes according to situation of primary tumor surgery (P < 0.001).

Supplementary Table S1. Demographic and clinical characteristics of metastatic breast cancer patients.

	HER2+/HoR+ N=1,293(%)	HER2-/HoR+ N=4,590(%)	HER2+/HoR- N=695(%)	HER2-/HoR- N=1,000(%)	Total N=7,578(%)	p-value[a]
Year of Diagnosis						0.477
2010	288(22.3)	1,082(23.6)	164(23.6)	257(25.7)	1,791(23.6)	
2011	317(24.5)	1,190(25.9)	167(24.0)	250(25.0)	1,924(25.4)	
2012	359(27.8)	1,151(25.1)	177(25.5)	241(24.1)	1,928(25.4)	
2013	329(25.4)	1,167(25.4)	187(26.9)	252(25.2)	1,935(25.5)	
Age						<0.001
<50	377(29.2)	962(21.0)	201(28.9)	266(26.6)	1,806(23.8)	
50-69	679(52.5)	2,366(51.5)	364(52.4)	520(52.0)	3,929(51.8)	
≥70	237(18.3)	1,262(27.5)	130(18.7)	214(21.4)	1,843(24.3)	
Race						<0.001
White	959(74.2)	3,543(77.2)	502(72.2)	666(66.6)	5,670(74.8)	
Black	226(17.5)	678(14.8)	120(17.3)	267(26.7)	1,291(17.0)	
Others[b]	102(7.9)	350(7.6)	70(10.1)	65(6.5)	587(7.7)	
Unknown	6(0.5)	19(0.4)	3(0.4)	2(0.2)	30(0.4)	
Insurance						0.296
Insured	1,199(92.7)	4,306(93.8)	660(95.0)	927(92.7)	7,092(93.6)	
Uninsured	69(5.3)	198(4.3)	26(3.7)	56(5.6)	349(4.6)	
Unknown	25(1.9)	86(1.9)	9(1.3)	17(1.7)	137(1.8)	
Marital status						0.079
Married	583(45.1)	2,020(44.0)	316(45.5)	422(42.2)	3,341(44.1)	
Not married[c]	643(49.7)	2,326(50.7)	329(47.3)	535(53.5)	3,833(50.6)	
Unknown	67(5.2)	244(5.3)	50(7.2)	43(4.3)	404(5.3)	
Tumor size(mm)						<0.001

≤20	154(11.9)	575(12.5)	81(11.7)	114(11.4)	924(12.2)	
21-50	504(39.0)	1,812(39.5)	239(34.4)	340(34.0)	2,895(38.2)	
> 50	461(35.7)	1,600(34.9)	285(41.0)	438(43.8)	2,784(36.7)	
Unknown	174(13.5)	603(13.1)	90(12.9)	108(10.8)	975(12.9)	
Positive regional nodes						<0.001
0	94(7.3)	195(4.2)	71(10.2）	97(9.7)	457(6.0)	
1-3	165(12.8)	572(12.5)	101(14.5）	162(16.2)	1,000(13.2)	
4-9	82(6.3)	359(7.8)	54(7.8）	78(7.8)	573(7.6)	
>10	80(6.2)	326(7.1)	47(6.8）	59(5.9)	512(6.8)	
No nodes examined	652(50.4)	2,504(54.6)	288(41.4）	441(44.1)	3,885(51.3)	
Unknown	220(17.0)	634(13.8)	134(19.3）	163(16.3)	1,151(15.2)	
Grade						<0.001
I	22(1.7)	456(9.9)	4(0.6)	14(1.4)	496(6.5)	
II	442(34.2)	2,030(44.2)	162(23.3)	157(15.7)	2,791(36.8)	
III and UD[d]	694(53.7)	1,460(31.8)	457(65.8)	741(74.1)	3,352(44.2)	
Unknown	135(10.4)	644(14.0)	72(10.4)	88(8.8)	939(12.4)	
Surgery						<0.001
Yes	496(38.4)	1,569(34.2)	295(42.4)	466(46.6)	2,826(37.3)	
No	789(61.0)	3,007(65.5)	394(56.7)	531(53.1)	4,721(62.3)	
Unknown	8(0.6)	14(0.3)	6(0.9)	3(0.3)	31(0.4)	
Radiation						0.054
Yes	434(33.6)	1,612(35.1)	225(32.4)	342(34.2)	2,613(34.5)	
No	803(62.1)	2,848(62.0)	442(63.6)	633(63.3)	4,726(62.4)	
Unknown	56(4.3)	130(2.8)	28(4.0)	25(2.5)	239(3.2)	
Single metastasis						<0.001
Bone	417(32.3)	2,189(47.7)	126(18.1)	221(22.1)	2,953(39.0)	

Brain	11(0.9)	33(0.7)	12(1.7)	26(2.6)	82(1.1)
Liver	139(10.8)	221(4.8)	115(16.5)	97(9.7)	572(7.5)
Lung	117(9.0)	302(6.6)	99(14.2)	170(17.0)	688(9.1)
Unknown or multiple metastasis	609(47.1)	1,845(40.2)	343(49.4)	486(48.6)	3,283(43.3)

Abbreviations: UD, undifferentiated; HER2, human epidermal growth factor receptor-2; HoR, hormone receptor.

a. p-value was assessed using the Pearson's $\chi 2$ test.

b. Including American Indian/Alaskan native and Asian/Pacific Islander.

c. Including divorced, separated, single (never married), and widowed.

d. Including grade 3 and undifferentiated.

Supplementary Table S2. Multivariate regression model analysis of overall survival and breast cancer-specific survival of stage IV patients with single metastasis information.

	Overall survival		Breast cancer-specific survival	
	HR(95% CI)	P-value	HR(95% CI)	P-value
Single Metastasis				
bone	Reference	-	Reference	-
brain	2.57(1.92-3.44)	<0.001	2.71(2.01-3.66)	<0.001
liver	1.40(1.20-1.65)	<0.001	1.45(1.22-1.71)	<0.001
lung	1.00(0.86-1.16)	0.988	1.00(0.85-1.17)	0.978
Year of Diagnosis				
2010	Reference	-	Reference	-
2011	0.93(0.82-1.06)	0.282	0.96(0.84-1.10)	0.561
2012	0.90(0.77-1.05)	0.169	0.92(0.78-1.09)	0.330
2013	0.79(0.63-0.99)	0.043	0.82(0.64-1.04)	0.094
Age				
<50	Reference	-	Reference	-
50-69	1.42(1.22-1.65)	<0.001	1.38(1.18-1.61)	<0.001
≥70	2.40(2.03-2.82)	<0.001	2.19(1.84-2.60)	<0.001
Race				
White	Reference	-	Reference	-
Black	1.20(1.04-1.38)	0.011	1.15(0.99-1.33)	0.066
Others[a]	1.00(0.79-1.26)	0.988	1.01(0.79-1.28)	0.965
Insurance				
Insured	Reference	-	Reference	-
Uninsured	1.57(1.23-2.03)	<0.001	1.68(1.30-2.16)	<0.001

Marital status				
Married	Reference	-	Reference	-
Not married[b]	1.30(1.16-1.46)	<0.001	1.28(1.13-1.45)	<0.001
Tumor size(mm)				
≤20	Reference	-	Reference	-
21-50	0.96(0.80-1.15)	0.639	0.97(0.80-1.17)	0.740
>50	1.09(0.91-1.30)	0.354	1.17(0.97-1.41)	0.110
Regional nodes positive				
0	Reference	-	Reference	-
1-3	1.10(0.83-1.45)	0.530	1.09(0.81-1.47)	0.578
4-9	1.29(0.95-1.76)	0.110	1.30(0.94-1.80)	0.113
>10	1.40(1.02-1.92)	0.035	1.45(1.04-2.01)	0.028
No nodes exmamined	1.57(1.19-2.05)	0.001	1.57(1.18-2.09)	0.002
Grade				
I	Reference	-	Reference	-
II	1.10(0.86-1.39)	0.455	1.15(0.89-1.49)	0.274
III and UD[c]	1.58(1.25-2.01)	<0.001	1.69(1.30-2.19)	<0.001
Molecular Subtype				
HER2+/HoR+	0.77(0.65-0.92)	0.004	0.77(0.64-0.92)	0.005
HER2-/HoR+	Reference	-	Reference	-
HER2+/HoR-	1.18(0.96-1.46)	0.119	1.16(0.93-1.45)	0.176
HER2-/HoR-	2.82(2.42-3.28)	<0.001	2.87(2.44-3.36)	<0.001
Surgery				
Yes	Reference	-	Reference	-
No	1.66(1.41-1.96)	<0.001	1.67(1.40-1.99)	<0.001
Radiation				

Yes	Reference	-	Reference	-
No	1.19(1.05-1.34)	0.006	1.18(1.04-1.35)	0.011

Abbreviations: HR, hazard rate; CI, confidence interval; UD, undifferentiated; HER2, human epidermal growth factor receptor-2; HoR, hormone receptor.

We hid the unknown in order to avoid data confusion.

a. Including American Indian/Alaskan native and Asian/Pacific Islander.

b. Including divorced, separated, single (never married), and widowed.

c. Including grade 3 and undifferentiated.
